# AHP DB: a reference database of proteins in the human aqueous humor

**DOI:** 10.1093/database/baae001

**Published:** 2024-01-29

**Authors:** Tae Jin Lee, Arnav Goyal, Garrett Jones, Joshua Glass, Vishal Doshi, Kathryn Bollinger, Lane Ulrich, Saleh Ahmed, Sai Karthik Kodeboyina, Amy Estes, Marc Töteberg-Harms, Wenbo Zhi, Shruti Sharma, Ashok Sharma

**Affiliations:** Center for Biotechnology and Genomic Medicine, Medical College of Georgia, Augusta University, 1120 15th Street, Augusta, GA 30912, USA; Center for Biotechnology and Genomic Medicine, Medical College of Georgia, Augusta University, 1120 15th Street, Augusta, GA 30912, USA; Center for Biotechnology and Genomic Medicine, Medical College of Georgia, Augusta University, 1120 15th Street, Augusta, GA 30912, USA; Center for Biotechnology and Genomic Medicine, Medical College of Georgia, Augusta University, 1120 15th Street, Augusta, GA 30912, USA; Center for Biotechnology and Genomic Medicine, Medical College of Georgia, Augusta University, 1120 15th Street, Augusta, GA 30912, USA; Department of Ophthalmology, Medical College of Georgia, Augusta University, 1120 15th Street, Augusta, GA 30912, USA; Department of Ophthalmology, Medical College of Georgia, Augusta University, 1120 15th Street, Augusta, GA 30912, USA; Center for Biotechnology and Genomic Medicine, Medical College of Georgia, Augusta University, 1120 15th Street, Augusta, GA 30912, USA; Center for Biotechnology and Genomic Medicine, Medical College of Georgia, Augusta University, 1120 15th Street, Augusta, GA 30912, USA; Mass General Brigham, 399 Revolution Drive, Somerville, MA 02145, USA; Department of Ophthalmology, Medical College of Georgia, Augusta University, 1120 15th Street, Augusta, GA 30912, USA; Department of Ophthalmology, Medical College of Georgia, Augusta University, 1120 15th Street, Augusta, GA 30912, USA; Center for Biotechnology and Genomic Medicine, Medical College of Georgia, Augusta University, 1120 15th Street, Augusta, GA 30912, USA; Center for Biotechnology and Genomic Medicine, Medical College of Georgia, Augusta University, 1120 15th Street, Augusta, GA 30912, USA; Department of Ophthalmology, Medical College of Georgia, Augusta University, 1120 15th Street, Augusta, GA 30912, USA; Center for Biotechnology and Genomic Medicine, Medical College of Georgia, Augusta University, 1120 15th Street, Augusta, GA 30912, USA; Department of Ophthalmology, Medical College of Georgia, Augusta University, 1120 15th Street, Augusta, GA 30912, USA; Department of Population Health Sciences, Medical College of Georgia, Augusta University, 1120 15th Street, Augusta, GA 30912, USA

## Abstract

The aqueous humor (AH) is a low-viscosity biofluid that continuously circulates from the posterior chamber to the anterior chamber of the eye. Recent advances in high-resolution mass-spectrometry workflows have facilitated the study of proteomic content in small-volume biofluids like AH, highlighting the potential clinical implications of the AH proteome. Nevertheless, in-depth investigations into the role of AH proteins in ocular diseases have encountered challenges due to limited accessibility to these workflows, difficulties in large-scale AH sample collection and the absence of a reference AH proteomic database. In response to these obstacles, and to promote further research on the involvement of AH proteins in ocular physiology and pathology, we have developed the web-based Aqueous Humor Proteomics Database (AHP DB). The current version of AHP DB contains proteomic data from 307 human AH samples, which were analyzed using liquid chromatography-tandem mass spectrometry (LC-MS/MS). The database offers comprehensive information on 1683 proteins identified in the AH samples. Furthermore, relevant clinical data are provided for each analyzed sample. Researchers also have the option to download these datasets individually for offline use, rendering it a valuable resource for the scientific community.

**Database URL**: https://ahp.augusta.edu/

## Introduction

The aqueous humor (AH) is a transparent, low-viscosity fluid produced by the ciliary body epithelium, circulating from the posterior to the anterior chamber of the eye (1). As a multifunctional biofluid, the AH plays essential roles in supplying nutrients, removing waste and providing oxygen to the avascular tissues of the ocular environment (2, 3). Homeostatic balance is achieved through continuous drainage and secretion, ensuring a steady flow that replenishes depleted nutrients and regulates intraocular pressure (IOP) within optimal physiological limits (4–6).

The proteomic composition of the AH is crucial for various cellular processes, including cell-to-cell communication, signal transduction, immunological modulation and cell proliferation (7–9). Consequently, researchers have investigated changes within the AH proteome associated with different pathophysiological conditions (7, 8, 10–26). These investigations have led to evidence linking specific AH proteins to ocular diseases, such as cataracts (12, 15, 19, 23), glaucoma (15, 18, 21, 27), age-related macular degeneration (20, 22), uveitis (28, 29), retinoblastoma (30, 31) and diabetic retinopathy (17, 24). Moreover, the dynamics of the AH proteome have been observed to vary across demographic variables, such as sex (13) and race (32), with certain proteins involved in inflammatory protection and immune response found to be more abundant in AH samples from female and African American cohorts, respectively. These discoveries underscore the importance of further exploring the AH proteome and the potential to unveil novel molecular mechanisms.

Given that the AH is a small-volume biofluid with low protein concentration, reproducibility in proteomic profiling of the AH has been limited, as detecting low-abundance proteins can be inconsistent or potentially masked by more abundant molecules, such as transferrin, albumin and immunoglobulins (13, 33–36). Liquid chromatography-tandem mass spectrometry (LC-MS/MS) has emerged as a valuable analytical platform for identifying and quantifying a wide range of proteins in complex, low-volume biofluids, including tears, cerebrospinal fluid, saliva and the AH (7, 8, 10–12, 15, 16, 27, 33, 37–42). The exceptional sensitivity, high-throughput capabilities and reliable detection of lower-abundance proteins have significantly expanded the known AH proteome (33, 43).

In this study, we analyzed 307 AH samples using LC-MS/MS and developed a web-based resource known as the ‘Aqueous Humor Proteomics Database’ (AHP DB). This reference database of commonly identified AH proteins aims to provide valuable insights to the vision research community, helping them to better understand physiological and pathological proteomic signatures within the AH.

## Materials and methods

### Human subjects and sample collection

Participants in this study were recruited from patients undergoing cataract or glaucoma surgical procedures at the Augusta University Medical Center. Written, informed consent was obtained from all subjects recruited, and this study was conducted in accordance with the Declaration of Helsinki and approved by the Institutional Review Board of Augusta University (IRB# 611480). Following paracentesis incision into the anterior chamber, the AH was drained using a cortical cleaving hydrodissector (585157, Beaver-Visitec International, Waltham, MA, USA). Typical AH sample volumes ranged from 50 to 200 µL depending on the depth of the anterior chamber. The AH sample was immediately transferred to a 1.7 mL Eppendorf tube and stored at −80°C until further analysis. These methods were safe for all subjects and did not affect the duration of the surgery. Following sample collection, a chart review was conducted to obtain demographic and clinical information pertaining to the study participants.

### AH sample preparation and LC-MS/MS analysis

The AH samples were lyophilized and reconstituted in 30 μL of 8 m urea in 50 mM Tris-HCl buffer at pH 8.0. To minimize interference with proteomic mapping, cysteine residues in each sample were reduced and alkylated using 20 mM DTT and 55 mM iodoacetamide, respectively. Subsequently, 240 μL of 50 mM ammonium bicarbonate buffer was added to each sample to lower the urea concentration to <1 m. Total protein concentration was measured using a Bradford assay kit, following the manufacturer’s instructions.

For LC/MS-MS preparation, the samples were digested with trypsin at a 1:20 (*w*/*w*) at 37°C overnight. After trypsin digestion, the samples were cleaned using a C18 spin plate and then separated and analyzed via the Ultimate 3000 nano-UPLC system and Orbitrap Fusion Tribrid mass spectrometer. The reconstituted peptide mixture (6 μL) was trapped and washed on a Pepman100 C18 spin plate using a gradient of 2% acetonitrile in water and 0.1% formic acid for 10 min at a flow rate of 20 μL/min. Subsequently, the samples were separated on a Pepman100 RSCLC C18 column using a gradient of 2–40% acetonitrile in water and 0.1% formic acid at a flow rate of 300 nL/min for 120 min.

The eluted peptides were subjected to nano-electrospray ionization at 275°C and 2000 V. The peptides were then analyzed in the Orbitrap Fusion Tribrid mass spectrometer using data-dependent acquisition in positive mode. The parameters for analysis included 120 000 FWHM from 300 to 1500 m/z, ion-trap MS analyzer in top speed mode at a 2 s cycle time and dynamic exclusion settings with the repeat count set to 1, repeat duration set to 15 s and exclusion duration set to 30 s. Collision-induced dissociation (CID) fragmentation was used with normal collision energy set to 30%.

### Protein identification and quantification

Proteome Discoverer (version 2.2, Thermo Fisher Scientific, Waltham, MA, USA) software was utilized to process raw MS files for protein identification and quantification. The SequestHT algorithm was employed to align identified peptide sequences to the UniProt-SwissProt database, with parameters set for 10 ppm precursor mass tolerance, 0.6 Da product ion tolerance, static carbidomethylation for cysteine (+57.021 Da), dynamic oxidation for methionine (+15.995 Da) and dynamic phosphorylation for serine, threonine and tyrosine (+79.966 Da). Proteins containing similar peptide sequences that could not be differentiated by LC-MS/MS were instead grouped based on the principles of parsimony. The peptide-spectrum match (PSM) count of each protein was used as a semi-quantitative measure of relative protein expression in AH.

### Database and website development

AHP DB was developed on a Microsoft platform using Asp.net for web development with JavaScript to create a highly interactive web interface. Microsoft SQL Server was used for data storage, with the website hosted on an Internet Information Services (IIS) web server. Data generated from LC-MS/MS analyses were uploaded to the server via the web interface. AHP DB was designed with internal programming to process the data files, generate appropriate tables and add data records into the database. User-friendly filtering and search options for each data column were also integrated. Clinical data corresponding to each sample were collected and updated in a Microsoft Excel file, which was subsequently uploaded to the database. The website was designed to automatically update all database pages and downloadable data sets following each upload.

## Results

### Database contents

The AHP DB serves as an accessible public resource for the collection and reference of AH proteomic data, which is shared with the research community. The user interface developed for the AHP DB allows users to access each component of the database via the following tabs in the navigation bar at the top of each page: ‘Home’, ‘Protein Summary’, ‘Protein Data’, ‘Clinical Data’ and ‘Data Download’.

The ‘Home’ tab brings the user to the front page of the database, containing a brief description of both the importance of AH and the technologies used to collect and analyze AH proteins for the database. The current version of the database contains 1683 proteins detected in >5% of AH samples from 307 subjects.

**Figure 1.**
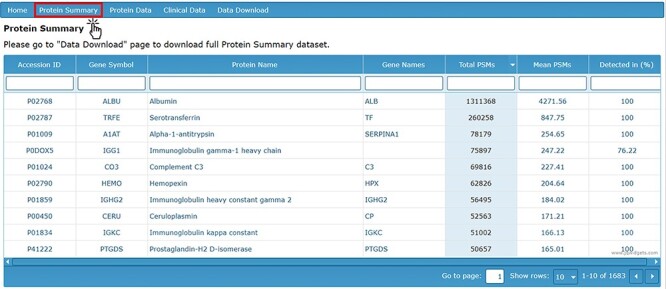
Protein Summary page containing accession ID, gene symbol, protein name, gene names, PSM counts and detection level.

The ‘Protein Summary’ tab displays a table of all identified proteins accompanied by the total number and mean number of PSMs for each protein, as well as the percentage of AH samples in which each protein was detected (Figure 1). Based on the data collected to date, the 50 most abundant proteins detected in the AH are listed in Table 1. The top 50 most abundant proteins are defined based on their detection levels (in at least 50% of samples) and higher mean PSM counts.

**Table 1.** Top 50 proteins identified in human aqueous humor

Serial no.	Uniprot ID	Gene symbol	Protein name	Mean PSM	Samples Detected In (%)
1	P02768	ALBU	Albumin	4271.56	100
2	P02787	TRFE	Serotransferrin	847.75	100
3	P01009	A1AT	Alpha-1-antitrypsin	254.65	100
4	P0DOX5	IGG1	Immunoglobulin gamma-1 heavy chain	247.22	76.22
5	P01024	CO3	Complement C3	227.41	100
6	P02790	HEMO	Hemopexin	204.64	100
7	P01859	IGHG2	Immunoglobulin heavy constant gamma 2	184.02	100
8	P00450	CERU	Ceruloplasmin	171.21	100
9	P01834	IGKC	Immunoglobulin kappa constant	166.13	100
10	P41222	PTGDS	Prostaglandin-H2 D-isomerase	165.01	100
11	P02766	TTHY	Transthyretin	160.76	100
12	P36955	PEDF	Pigment epithelium-derived factor	158.89	100
13	P10745	RET3	Retinol-binding protein 3	156.18	99.35
14	P02763	A1AG1	Alpha-1-acid glycoprotein 1	141.03	100
15	P01860	IGHG3	Immunoglobulin heavy constant gamma 3	140.51	100
16	P0C0L5	CO4B	Complement C4-B	135.16	96.74
17	P0DOX7	IGK	Immunoglobulin kappa light chain	113.8	100
18	P0C0L4	CO4A	Complement C4-A	113.22	80.13
19	P02774	VTDB	Vitamin D-binding protein	112.03	100
20	P01861	IGHG4	Immunoglobulin heavy constant gamma 4	105.76	97.39
21	P02647	APOA1	Apolipoprotein A-I	100.74	100
22	P10909	CLUS	Clusterin	96.09	100
23	P0DOY2	IGLC2	Immunoglobulin lambda constant 2	83.8	98.05
24	P01034	CYTC	Cystatin-C	82.54	99.67
25	P19652	A1AG2	Alpha-1-acid glycoprotein 2	82.43	100
26	P01023	A2MG	Alpha-2-macroglobulin	73.1	100
27	P02765	FETUA	Alpha-2-HS-glycoprotein	70	100
28	P04217	A1BG	Alpha-1B-glycoprotein	66.48	100
29	P68871	HBB	Hemoglobin subunit beta	63.86	52.44
30	P22352	GPX3	Glutathione peroxidase 3	62.06	99.67
31	P01008	ANT3	Antithrombin-III	61.99	100
32	P02749	APOH	Beta-2-glycoprotein 1	58	100
33	P06396	GELS	Gelsolin	57.78	100
34	P01876	IGHA1	Immunoglobulin heavy constant alpha 1	56.43	99.35
35	Q9UBP4	DKK3	Dickkopf-related protein 3	54.2	99.67
36	P00751	CFAB	Complement factor B	53	100
37	P01011	AACT	Alpha-1-antichymotrypsin	49.49	100
38	Q13822	ENPP2	Ectonucleotide pyrophosphatase/phosphodiesterase family member 2	49.38	99.67
39	P00738	HPT	Haptoglobin	47.52	97.07
40	Q12805	FBLN3	EGF-containing fibulin-like extracellular matrix protein 1	47.28	100
41	P0DOX8	IGL1	Immunoglobulin lambda-1 light chain	45.77	64.17
42	P00747	PLMN	Plasminogen	44.16	100
43	Q9UBM4	OPT	Opticin	38.5	99.67
44	P07339	CATD	Cathepsin D	36.4	99.35
45	P25311	ZA2G	Plasma protease C1 inhibitor	35.55	100
46	P04264	K2C1	Zinc-alpha-2-glycoprotein	33.56	100
47	P06727	APOA4	Keratin, type II cytoskeletal 1	33.24	71.34
48	P00734	THRB	Apolipoprotein A-IV	32.5	98.7
49	P07998	RNAS1	Prothrombin	31.88	100
50	P25311	ZA2G	Ribonuclease pancreatic	31.44	99.67

**Figure 2.**
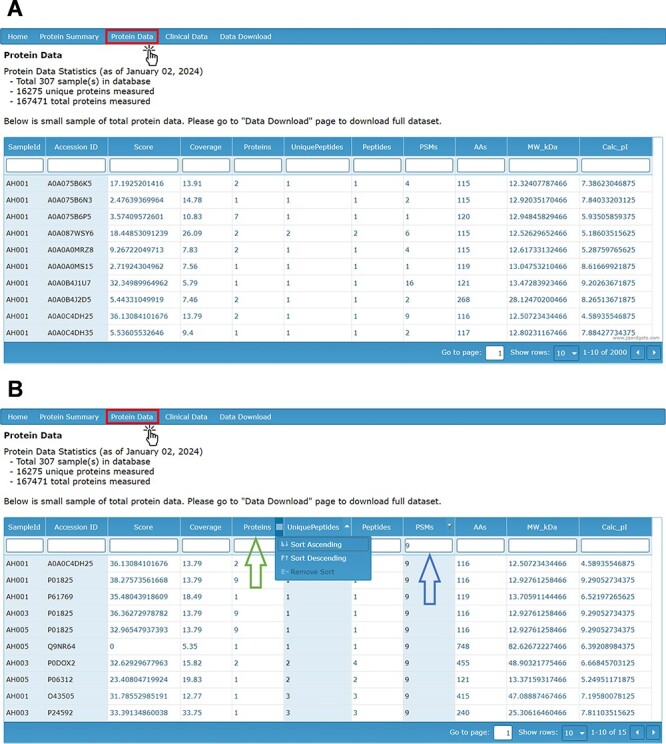
(A) Protein Data page providing visual display of LC-MS/MS analysis of proteins by sample ID and with (B) filters applied by search (right arrow) and by sorting ascending or descending (left arrow).

In the database, proteomic data can be accessed through the ‘Protein Data’ tab, which provides a visual display of the mass spectrometry output data for all identified proteins (Figure 2A). These raw data are also available for download in a separate file. The data are stratified by sample ID and detail the UniProt ID (accession number), score, coverage, number of identified proteins in the protein group, number of unique peptides, number of peptides, PSM count, number of amino acids, protein molecular weight and calculated isoelectric point (pI).

The score of the associated protein reflects the sum of ion scores corresponding to the peptides identified, providing a measure of the confidence in protein identification. The coverage column refers to the proportion of the total protein sequence that could be matched by identified peptides. The PSM count refers to the number of PSMs for each peptide collected and identified by LC-MS/MS Factors such as run time, protein concentration, and sample variation may influence the total PSM count for any individual protein. The number of proteins refers to the number of protein groups that include the identified protein. The number of unique peptides denotes the number of peptide fragments that uniquely belong to one protein group. The number of peptides indicates the total number of peptide sequences covering a protein group. The design of the database allows each of these column items to be filtered by a search bar and sorted in ascending or descending order (Figure 2B).

**Figure 3.**
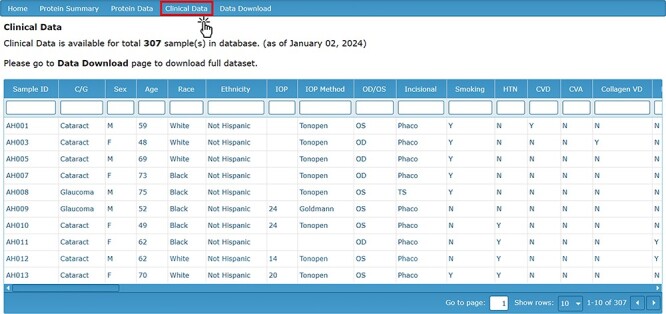
Clinical Data page detailing the clinical characteristics and demographic information for each sample.

To complement the proteomic contents of the AHP DB, a reference clinical data set is also available for the analysis of the proteomic data in the context of clinical and demographic characteristics. The ‘Clinical Data’ tab displays information about the subjects from whom the AH samples were collected. This includes relevant ocular pathology (cataract or glaucoma), demographic information (sex, age, race and ethnicity), ocular characteristics (IOP, cup area, disc area, etc.), comorbidities, current medications and other clinical characteristics (Figure 3).

**Figure 4.**
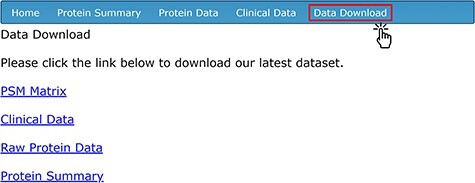
Data download options for protein and clinical data sets.

To provide easy access to all of the data for secondary analysis, four separate data sets related to the compiled AH proteomic studies are available for download on the ‘Data Download’ page (Figure 4). To access the download contents, users need to create an account with a unique username and password through the ‘sign-up’ page. The ‘PSM Matrix’ link downloads a file containing the PSM counts of each protein detected via LC-MS/MS, stratified by de-identified sample ID. The UniProt ID (labeled as ‘Accession’), total PSM count among all samples and sample-by-sample PSM count are available for each protein. The ‘Clinical Data’ link downloads a CSV file containing all of the clinical and demographic information corresponding to each sample; this is the same information that is found in the ‘Clinical Data’ tab. The ‘Raw Protein Data’ link downloads a file that includes the full mass spectrometry output generated by Proteome Discoverer using the SequestHT algorithm. The data on the database’s ‘Protein Summary’ tab are available for download on the ‘Data Download’ page as a CSV file by clicking on the ‘Protein Summary’ link.

### Data search and retrieval

To query information on any of the data display pages, the user can enter a character string into the search bar directly beneath the title of the chosen column. The data set will automatically be filtered to display content containing the desired search term. Additionally, users can input search terms into multiple columns simultaneously. Users can also sort through all displayed data sets using the drop-down menu located on the far right of the associated column name. Each data set within the database can be sorted by column in either ascending or descending order. Furthermore, users can filter and sort the data simultaneously in any visual data set on the database webpage. An example of these functions on the ‘Protein Data’ page is shown in Figure 2B.

## Discussion

AH plays a critical role in maintaining IOP and ocular homeostasis, relying on its diverse array of nutrients, electrolytes and protein content to modulate these functions. Characterizing the human AH proteome will further our understanding of the roles of specific molecules in health and enable more in-depth studies of their relation to pathogenesis. Due to interpersonal variation and the presence of low-abundance proteins, corroborating results can be challenging. Providing a reference database of the human AH proteome can supplement and guide interpretations of AH proteomics in future investigations of ocular pathology and physiology.

The AHP DB provides a publicly accessible collection of proteins identified in human AH using well-validated and highly sensitive mass spectrometry methods. AHP DB stores and presents proteomic information on human AH while concurrently offering protein summaries and corresponding clinical data. Subsets of the data compiled in this database have been analyzed and presented in our previous publications (32, 44, 45), shedding light on inter-population differences in the human AH proteome that may contribute to variations in ocular disease risk factors and incidence. As additional samples are collected and analyzed, the proteomic and corresponding clinical data will be incorporated into the database at regular intervals. In the future, we also plan to add functions that allow other users to upload their own AH proteomic data to the database.

Protein families in the AH can be analyzed to identify differences associated with demographic variables. In one previous study, the AH proteomic content of 88 subjects undergoing cataract surgery was analyzed to establish a preliminary overview of the human AH proteome. Notably, the levels of certain proteins, including SOD3 and TSPAN14, were found to differ significantly between African American and Caucasian subjects (32). Another prior study revealed sex- and race-specific differences among levels of apolipoproteins in the AH of subjects with primary open-angle glaucoma (45). Also, analysis of 49 samples from subjects with glaucoma revealed associations between the levels of several AH proteins and abnormal visual field parameters, demonstrating the clinical relevance of the AH proteome (44).

This database was generated using shotgun mass spectrometry, also referred to as bottom-up proteomics. Shotgun mass spectrometry represents a robust technique for the identification and quantification of proteins in complex biological samples. There are, however, inherent limitations to this methodology. Shotgun proteomics identifies peptides rather than intact proteins, potentially confounding protein identification in instances of multiple proteins sharing common peptide sequences. It is important to note that shotgun proteomics primarily offers relative quantification rather than absolute quantification. Additionally, shotgun proteomics using data-dependent acquisition prioritizes proteins present in higher abundance, thereby decreasing the reliability of detecting lower abundance proteins.

In conclusion, the current version of AHP DB offers a navigable interface featuring 1683 proteins detected in >5% of AH samples. Users can load and filter data according to clinical or proteomic profiles and subsequently download datasets for further analysis. AHP DB is a novel, freely accessible source of proteomic and corresponding clinical information about human AH. The database provides a user-friendly interface to enable researchers to analyze commonly expressed proteins from the human AH, thereby enhancing knowledge in the field of ophthalmology.
